# Assessing community knowledge, attitude and practices to strengthen communication strategy for Malaria Elimination Demonstration Project in Mandla

**DOI:** 10.1186/s12936-021-03884-y

**Published:** 2021-08-28

**Authors:** Harsh Rajvanshi, Kalyan B. Saha, Ravendra K. Sharma, Praveen K. Bharti, Sekh Nisar, Himanshu Jayswar, Ashok K. Mishra, Man Mohan Shukla, Aparup Das, Harpreet Kaur, Suman L. Wattal, Altaf A. Lal

**Affiliations:** 1 Malaria Elimination Demonstration Project, Mandla, Madhya Pradesh India; 2 Indian Council of Medical Research - National Institute of Research in Tribal Health (ICMR-NIRTH), Jabalpur, Madhya Pradesh India; 3 Directorate of Health Services, Government of Madhya Pradesh, Bhopal, India; 4 Indian Council of Medical Research, Department of Health Research, Ministry of Health and Family Welfare, New Delhi, India; 5 National Vector Borne Disease Control Programme, Ministry of Health and Family Welfare, New Delhi, India; 6 Foundation for Disease Elimination and Control of India, Mumbai, Maharashtra India

## Abstract

**Background:**

Changes in social, belief, and behavioural practices are essential for the success of any public health delivery programme. In the planning stages of the Malaria Elimination Demonstration Project (MEDP), priority was given to communication with a goal to develop capacity of health workers and to improve the knowledge, attitude and practices (KAP) of the people of Mandla. This paper describes the level of community knowledge on malaria, including its prevention, diagnosis, treatment-seeking behaviour, and the level of satisfaction with the services provided by the project.

**Methods:**

A cross sectional survey was undertaken in 1233 villages of Mandla to study the KAP and self-assessed improvement in knowledge and satisfaction level of the community. The goal of the study was to understand whether there is need for strengthening communication strategy of MEDP for better impact. The survey was conducted amongst the head/eligible members of the 733 households located in the nine blocks of the district using clustered random sampling.

**Results:**

Though four-fifths of the respondents were able to correlate the transmission of malaria with mosquitoes, misconceptions existed among them. The types of malaria were not known to everyone. Only 39% were aware of the Indoor Residual Spray (IRS) and 41% understood the value of Long-Lasting Insecticidal Nets (LLIN). Around 71% of subjects surveyed were aware of the proper diagnostic tests for malaria. A total of 87% of the respondents knew about the MEDP staff working in their respective villages.

**Conclusion:**

The study reported gaps in knowledge on malaria at community level. The self-assessment of the community revealed that the communication strategy established by MEDP in Mandla district has been useful to them as they are becoming better informed about the prevention and treatment aspects of disease. The lessons learned as revealed in the KAP survey will improve malaria elimination outcomes in a timely manner.

## Background

Mandla is predominantly socio-economically backward and a tribal district of Madhya Pradesh. In the year 2015, the district reported 3901 malaria cases (API—4.03) and was a category 3 district as per the National Strategic Plan 2017–2022 of the National Vector Borne Disease Control Programme. The data from 2015 was used for preparation of the operational plan for the Malaria Elimination Demonstration Project (MEDP) [1, 2].

MEDP is a public–private partnership between the Government of Madhya Pradesh, Indian Council of Medical Research through the National Institute of Research in Tribal Health (ICMR-NIRTH), and Foundation for Disease Elimination and Control of India (FDEC India), which was established by Sun Pharmaceutical Industries Ltd. as a not-for-profit entity. The project aimed at elimination of indigenous malaria transmission in the district using four principle strategies viz*.* (1) Robust surveillance and case management; (2) Vector control; (3) Information Education Communication and Behaviour Change Communication (IEC/BCC); and (4) Capacity building with continuous monitoring and learning [1]. The size and geographical terrain of Mandla, the endemicity of malaria, weak socio-economic status and the ability to retain field staff were the key challenges considered by the project at the onset of the study. These barriers have been well observed in studies conducted in other areas having similar geo-climatic and socio-cultural environment [3–8]. In the local tribal communities, the treatment-seeking behaviour is poor and help is sought only when the situation becomes serious. This community behaviour, which does not allow for rapid diagnosis and prompt treatment, is one of the risk factor for accomplishing the goals of malaria elimination of the National Vector Borne Disease Control Programme (NVBDCP).

In addition, the existing misconceptions about disease and prevention controls in the community and the treatment offered by the un-organized health care workers increases the risk of spread of malaria. For instance, the common belief in the community identifies mosquitoes as the primary mode for transmission of malaria, however, some also believe that insects other than mosquitoes, which increase in number during rainy season, also spread malaria [4]. Further, the acceptance of Long Lasting Insecticidal Nets (LLIN) is limited because of the perceived ill-effects and odour, which results in frequent washing that leads to reduction in the repellent capacity of the nets. Prior studies have shown that bed nets are at times used for fishing [4]. The fear of food contamination due to Indoor Residual Spraying (IRS) leads to its poor acceptance in the community [3–5].

The practice of black magic exists throughout the study district. In the tribal areas, it is a common belief that malaria is a punishment by the evil spirits. Due to immense faith on the supernatural powers of the traditional healers and their easy accessibility, the first-line of treatment is usually sought from these unqualified healers. They practice herbal medicine, faith healing, and symptomatic treatments [2, 4]. These practices are a threat to the programme driven anti-malarial services. Moreover, the frontline workers, particularly the over-committed Accredited Social and Health Activists (ASHA) are unable to fully comply with the NVBDCP guidelines and consider it the least priority work [4, 9].

In the planning stages of Malaria Elimination Demonstration Project (MEDP), emphasis was placed on developing a robust and effective IEC strategy. The communication strategy was developed “for the people and by the people”, which means that the communication tools were developed in consultation with the community and field staff. Eighteen months after starting the project, an assessment of the knowledge of malaria, its prevention, treatment seeking behaviour and the self-satisfaction level of the community was conducted. The lessons learned from these assessments not only improved the communication strategy, but also acted as a baseline to evaluate the project’s performance in the future.

The use of electronic and print media in the rural and tribal areas of Mandla district was very poor. Further, those who had access to these media channels were not sensitized enough to use it as a source of health education. The mobile connectivity was not uniform in rural areas of the district. Thus, the traditional communication strategy consisting of daily newspapers, radio, television and mobile phones was not sufficient to sensitize the masses. In the view of these limitations, the communication strategy was designed to have one-to-one interaction with the community to improve the knowledge and perception on preventive aspects and improving the anti-malarial services utilization factors among the population of Mandla. The communication strategy of MEDP involved: (1) inter-personal communication during door to door visit in study villages; (2) middle school children as agents of change; and (3) targeting weekly community markets (*Haat Bazars*) in the rural areas.

## Methods

### Communication strategy

**Fig. 1:**
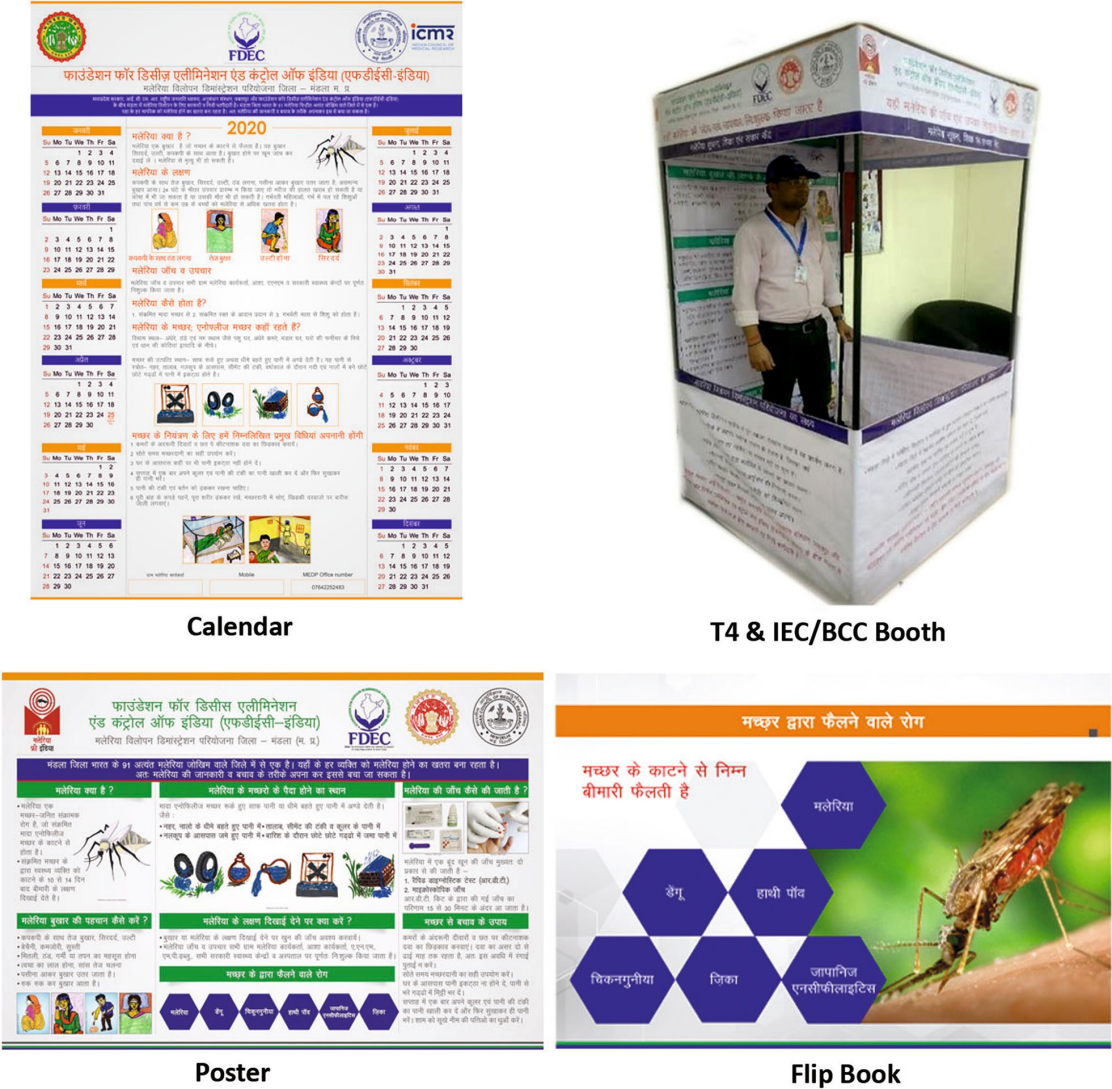
IEC BCC materials used by MEDP Mandla

The IEC-based communication strategy was developed and rolled out in collaboration with community members and MEDP field staff in 2017  (Fig. 1). Culture-based tools of communication were used for the IEC campaigns in MEDP. Lessons from prior studies done by ICMR-NIRTH in tribal areas were key drivers for selection of inter-personal communication as an appropriate IEC strategy for Mandla [4]. The strategy was based on the communication model used in malaria-endemic Baigachak area of Dindori district of Madhya Pradesh in 2013 by Saha et al*.* [5].

The key manpower strategy consisted of: (1) 235 Village Malaria Workers (VMWs) with higher secondary (10 + 2) educational level, who were locally recruited and trained to perform IEC activities in addition to active surveillance and case management; (2) 25 Malaria Field Coordinators (MFCs) with a minimum qualification of graduate (degree) level, who supervised the VMWs.

The IEC campaign consisted of: (1) Door-to-door communication at community level by using flip-books on malaria, which depicted pictographic information on malaria vectors, transmission mechanism, preventive, diagnosis and treatment aspects; (2) Specially designed malaria calendar was distributed in the places of public interest and assembly, such as *“Anganwadi Centres”* (Courtyard shelters) and “*Gram Panchyat Bhawans*” (Village council buildings). These calendars provided awareness messages in local language on transmission, prevention, diagnosis and treatment for malaria.; (3) Campaigns were conducted at middle school level, which included, exhibitions, quiz contests and popular lectures on malaria organized on regular basis; (4) Theatre-based activities including storytelling techniques were adopted; (5) School activities focussed on sensitizing the students and teachers on the misconceptions and informing them on the preventive and treatment aspects of malaria and empowering them as ‘agents of change’; and (6) IEC/T4 (Track fever, Test fever, Treat patient, Track patient) booths installed at rural weekly markets locally known as “*haat bazar*” (community markets). These weekly market were used for spot diagnosis and treatment for shoppers, who were febrile and approached the IEC booths.

### Survey

A cross-sectional survey was conducted in February 2019 to assess the communication strategy which was designed based on information available from other areas similar to Mandla and information available from Government websites.

### Survey instrument

A structured, pre-tested interview schedule was used. Self-assessment questions on knowledge and perception on malaria as well as on the effectiveness of MEDP staff and activities before and after initiation of the project was included in the survey.

### Sample size and sampling

The district has nine blocks and 25 clusters. Each cluster comprises of around 12 sub-centres. Each cluster had one MFC and 8–10 VMWs. This calculation ensured full coverage of entire district with active surveillance by the project staff on a weekly to fortnightly basis. Assuming a hypothesized outcome frequency of 50% with design effect of ‘2’ and 95% confidence interval in a population size of 1,150,000, the sample size was estimated to be 768. Using clustered random sampling, 30 random samples from each cluster were selected for interview. Non-response was also considered while interviewing the respondents. Either head or any eligible member from 773 households located in nine blocks of Mandla district was interviewed. The households were stratified in the villages and randomly selected.

### Quality control and statistical analysis

Twenty-five interviewers (MFCs) were trained by ICMR-NIRTH on survey techniques and tools. To ensure data precision and accuracy, random back-checks and spot-checks in 2% of the households were done. Survey schedules were entered in a format designed in CS PRO and later exported to IBM-SPSS-26 for Windows (IBM Corp., Armonk, NY) for data validation and analysis. In addition to descriptive statistics, adjusted odds ratio (AOR) through multivariate logistic regression were also attempted to understand the controlled effect of socio-economic determinants on variables of interest. Scaling technique was adopted to develop two dependent variables viz*.* (1) knowledge and attitude towards MEDP activities (on a 0–13 point scale); and (2) self-assessment of knowledge about malaria (on a 1–5 point scale) before and after the initiation of MEDP. These were correlated with independent variables such as age, sex, marital status, education, occupation, caste, income and wealth index (constructed).

## Results

### Household characteristics of study population

Out of the 773 household respondents interviewed, 67% were male and 33% were female. Most of the respondents were Scheduled Tribes (64%), followed by the Other Backward Castes (29%)—a collective term used by Government of India for disadvantaged castes, Scheduled Castes (6%), and General category (2%). Almost a quarter of the respondents (24%) had studied up-to primary school, followed by those with up-to middle school education (22%), and up-to high school or higher (34%). Around 19% of the respondents had not received any formal education.

Amongst the major occupational groups, half of the respondents worked as farmers, followed by unskilled labourers (28%), and around six percent were home makers. A very small proportion reported business (3%) and government service (2%) as means of livelihood. Only two percent were students. Around 44% of the respondents reported annual household income of INR 10,000 to 25,000 (USD 140 to 350). Fifteen percent and 20% mentioned INR 25,000 to 50,000 (USD 350 to 700) and INR 5,000 to 10,000 (USD 70 to 140), respectively. Some of the respondents (9%) could not ascertain their annual household income. The mean annual household income of the households in study area was INR 22,976 ± 1572 (USD 350 ± 22).

In terms of amenities and facilities, most of the houses were found to be “*kaccha*” (temporary mud houses) (68%), followed by “*pucca*” (brick and mortar houses) (20%) and “semi *pucca*” (temporary roof) (13%). Barring few, most of the households were electrified. Only 24% of the households had flush toilet facilities and another 23% had pit toilets. More than a quarter of household members used public toilets (31%) and open defecation (23%). Around 31% of the households had no vehicle to commute. Bicycle (44%) was an important means of transportation.

The main sources of drinking water were public hand pumps (50%), followed by public taps (10%). Unfiltered surface water from river, stream, pond, and lake was used by very few (2%) for drinking purposes. Overwhelming majority of them used wood (93%) as cooking fuel, followed mostly by liquid petroleum gas (39%) and dung cakes (16%). Around 78% of the households owned agricultural land.

### Perception on transmission of malaria

Nearly four-fifths of the respondents (78%) mentioned that malaria is transmitted by mosquitoes. However, misconception prevailed as 7% reported that malaria spreads by drinking dirty water, 1.3% reported transmission by eating stale food, 1.3% reported transmission by touching malaria infected persons, and also by other insect bites (1.1%). Around 11% did not recognize any means of transmission of malaria. Malaria was reported as a fatal disease by 85% of the respondents (Table 1).

**Table 1:** Knowledge on malaria transmission and its symptoms

	Frequency	Percentage
Agents of spread of malaria		
Mosquito bites	603	78.01
Drinking dirty water	56	7.24
Stale food	10	1.29
Touching infected person	10	1.29
Do not know	85	11
Other (ant bites, garbage etc.)	9	1.16
Site of mosquito breeding (multiple response possible)		
Dirty water	461	59.64
Garbage	139	17.98
Stagnant clean water	134	17.34
Muddy sludges/puddles	234	30.27
Do not know	80	10.35
Others	10	1.29
Symptoms of malaria (multiple response on symptoms is possible)		
Headache	228	29.5
Vomiting	74	9.57
High fever	226	29.24
Intermittent fever	31	4.01
Fever with chills	503	65.07
Do not know	129	16.69
Others	19	2.46
Types of malaria		
Vivax	8	1.03
Falciparum	12	1.55
Malariae	1	0.13
Ovale	20	2.59
Do not know	732	94.7
Information source for malaria		
ASHA	124	16.04
ANM	57	7.37
MTS/MI (GoMP)	6	0.78
VMW/MFC (MEDP)	579	74.9
Others	7	0.91
Total	773	100

### Perception on breeding sources of mosquitoes

It was a common understanding in the community that mosquitoes breed in water. It was found that around 60% of those surveyed reported that mosquitoes breed in dirty water, in garbage (18%), and in muddy sludges and puddles (30%). Around 10% did not have any understanding of the breeding places. Only 17% of the respondents mentioned stagnant clean water as the breeding place for malaria mosquitoes (Table 1).

### Perception on symptoms and type of malaria

Fever with headaches was the common symptom reported to identify malaria (65%). Around 17% had no knowledge on how to identify malaria. Almost all (95%) had no knowledge about the types of malaria. There were only a few (5.3%) who mentioned the parasite. Around three-fourth of the respondents mentioned MEDP field workers as the main source of information on the symptoms and type of malaria (Table 1).

### Perception on prevention

Efforts were made to assess the perception of the respondents on programmatic and non-programmatic preventive practices. Quite a number of respondents reported smoke by burning dry leaves of neem tree (*Azadirachta indica*) as the preferred way of prevention of mosquitoes, besides the use of commercially available mosquito repellent coils. Regarding programme-supported preventive measures, only 39% of the respondents reported awareness about Indoor Residual Spray (IRS) as a mosquito control measure. Only 52% respondents in this group could mention one or more government or non-government organisations responsible for IRS. Around 16% mentioned Government malaria workers and 10% reported MEDP field staff as the main source of information on IRS (Table 2). Around 41% of respondents had heard about LLINs. Most of them identified them as medicated nets, while some reported it as Government nets, insecticidal nets etc. Amongst them, 83% knew its importance in malaria control. The correct distinction between LLIN and ordinary net was made by 64% of the respondents. Furthermore, correct mechanism of usage and washing was known to 80% and 36% of the respondents, respectively. Though 95% of them mentioned its use mainly to avoid mosquitoes, three percent reported using it to protect from other insects and snakes. A small number (4%) also used it as fishing nets (Table 2). Around 41% knew about the high-risk groups for malaria. Seventy percent mentioned new-borns as most susceptible followed by the elderly (25%) and pregnant women (24%). Only few knew the names of the anti-malarial drugs (Table 2). The perception on high risk group is also considered as an indicator of preventive approach necessary for protecting the vulnerable section of the community.

**Table 2:** Knowledge on programme driven preventive measures

	Frequency		Percentage
Knowledge on indoor residual spray	300 (out of 773)		38.8
Among those who know IRS (N = 300)			
Know its importance	227		75.7
Do you know how it is done	265		88.3
Who does it	176		58.7
Source of knowledge			
Total		300	
GoMP staff	47		15.7
MEDP staff	31		10.3
Others	77		25.7
Do not know	145		48.3
Total	300		100
Knowledge on long lasting insecticidal nets (LLIN)			
Heard about LLINs	318 (out of 773)		41.1
LLIN was known to community as (N = 318)			
Medicated nets	124		39.0
Govt nets	69		21.7
Insecticide nets	12		3.8
LLIN	19		6.0
Others	94		29.6
Know the importance	265		83.3
Know the difference between regular and LLIN	203		63.8
Knowledge of fixing the nets	255		80.2
Knowledge of washing and drying nets	115		36.2
Purpose for which LLINs are being used			
Fishing	11		3.5
Protection from mosquitoes	303		95.3
Protection from insects and snakes	8		2.5
Total	318		
Know high risk groups for malaria		320 (out of 773)	41.4
Risk groups (multiple responsible) (N = 320)			
Pregnant women	78		24.4
New born	223		69.7
Age more than 60 years	80		25.0
Labour/migratory workers	20		6.3
Do not know	19		5.9
Know the names of drugs for malaria	45 (out of 773)		5.8
Main drugs for malaria (multiple response)			
Chloroquine	39		5.0
Primaquine	11		1.4
ACT	3		0.4

### Perception on diagnosis

Regarding knowledge about malaria diagnosis, almost everyone knew the requirement of blood specimen for testing, but only 71% (550) were aware of the Rapid Diagnostic Tests(RDTs) or the blood slides as the diagnostic tests. Amongst them, 48% of the respondents were aware of the RDTs and 52% mentioned blood slide examination as the diagnostic method (Table 3).

**Table 3:** Knowledge and practices followed in the community related to malaria diagnosis and treatment

	Frequency	Percentage
1st Line of consultation for diagnosis and treatment for fever (N = 773)		
Priest	30	3.9
Gunia	16	2.1
Traditional Medicine Practitioners	45	5.8
Faith Healers	23	3.0
ASHA/ANM	141	18.2
MEDP	309	40.0
SC/PHC/CHC/District Hospital	616	79.7
Pharmacist/Private Practitioners	106	13.7
Knowledge on diagnostic tests for malaria	**550 (Out of 773)**	**71.2**
Those who knows diagnostic tests (N = 550)		
RDT	266	48.4
Blood slide examination	284	51.6
Nature of bio sample used for diagnostic test		
Blood	546	99.3
Do not know	4	0.7
Total	550	
Fever cases reported		
Any fever case in family in the past six months?	**326 (Out of 773)**	42.2
Blood test done for these fever cases?	232 (Out of 326)	71.2
Source where diagnosis conducted (N = 232)		
Traditional medicine practitioners	1	0.3
ASHA/ANM/MTS/MI	16	6.9
VMW (MEDP)	161	69.4
SC/PHC/CHC/DH	53	22.8
Pharmacist/Private Practitioner	19	8.2
Expenditure on the blood test (N = 232)		
FREE	208	89.7
10–50 INR	7	3.0
100–200 INR	5	2.2
200–500 INR	3	1.3
50–100 INR	8	3.4
500–1000 INR	1	0.4
Result of the blood test		
Result informed after the test	188 (Out of 232)	81.0
Negative	167 (Out of 188)	88.8
Positive	10 (Out of 188)	5.3
Do not remember	11 (Out of 188)	5.9
Expenditure on treatment (13 respondents could remember)		
FREE	8	61.5
50–100 INR	1	7.7
200–500 INR	2	15.4
500–1000 INR	2	15.4

Around 42% (326) of the respondents reported a case of fever in their household during six months preceding the survey and amongst them, 71% (232) mentioned that the patient had gone for blood testing mostly from MEDP field staff (69%), followed by visit to government health posts (23%), and chemist shop/ private practitioners (8%). Some of the patients availed services from multiple sources for testing of blood. Overwhelming majority of the households (90%) availed these services free of cost. Those who spent on diagnosis, the mean cost amounts to INR 140 ± 161 (USD 2 ± 2.5) per blood test. Around 81% (188) of the households were informed about the test results and 5% (10) amongst them were reported positive for malaria, and another 6% (11) could not recall the result at the time of the interview.

### Perception on treatment

In the event of fever, it was found that the community was consulting multiple health care providers. The first-line for treatment were Government health facilities (80%), followed by MEDP field staff (40%), programme-driven Accredited Social Health Activists (ASHA)/ Auxiliary Nurse Midwife (ANM) (18%), and chemist shops (14%). However, around 15% of the respondents reported that community members also visited priests, herbal healers and faith healers for seeking treatment for fever (Table 3). The malaria-specific treatment was provided free-of-cost by the Government and MEDP.

### Factors associated with knowledge on transmission, symptoms, programme driven preventive measures and diagnosis

Though 78% of the respondents correctly reported that mosquito is responsible for the spread of malaria, the logistic regression model revealed an inverse relationship between age of the respondents and their knowledge that mosquito is responsible for the spread of malaria. Females and unmarried respondents were better aware of the transmission. Education was significantly associated with the correct knowledge of mosquito as transmission agent (AOR: 3.78, p = 0.001) and its breeding site (AOR: 8.97, p = 0.001). Further, it was observed that compared to the scheduled tribes and scheduled castes, other social groups were better aware of the transmission agent. No similar association could be established with occupation and economic condition (Table 4).

**Table 4:** Logistic regression results showing factors associated with correct knowledge on malaria transmission, symptoms, breeding sites, programme sponsored preventive measures and malaria diagnosis tests

Variables	N	Knowledge of malaria transmission and breeding site of mosquitoes		Knowledge on main symptom of malaria and reported programme sponsored preventive measures		Knowledge on malaria diagnosis tests
		Malaria transmits by mosquito bite = 78.4% (AOR)	Breeding site of mosquito (stagnant clean water = 17.3%) (AOR)	Symptom of malaria (Fever with chill = 65.1%) (AOR)	Prevention of malaria (use of LLIN/IRS = 73.5%) (AOR)	Diagnosis of malaria (by RDT/Blood Slide) = 70.6%) (AOR)
Age						
18–29 30–44 45 +	246 262 265	– 0.84 (0.50–1.42) 0.69 (0.40–1.17)	– 1.24 (0.72–2.12) 1.43 (0.77–2.66)	– 1.52 (1.01–2.28)* 2.80 (1.76–4.45)***	– 0.90 (0.56–1.46) 0.73 (0.44–1.21)	– 1.51 (0.97–2.36) 1.47 (0.91–2.37)
Sex						
Female Male	256 517	– 0.97 (0.64–1.49)	– 0.85 (0.53–1.36)	– 0.95 (0.66–1.36)	– 0.91 (0.61–1.33)	– 1.31 (0.90–1.89)
Marital status						
Unmarried Married	107 666	– 0.99 (0.52–1.90)	– 0.67 (0.37–1.22)	– 0.71 (0.42–1.19)	– 2.18 (1.27–3.76)**	– 1.06 (0.62 -1.83)
Education						
Illiterate Primary Middle High school	147 188 170 268	– 1.72 (1.05–2.83)* 2.34 (1.34–4.08)*** 3.78 (2.09–6.86)***	– 2.21 (0.88–5.59) 4.48 (1.79–11.17)** 8.97 (3.65–22.04)***	– 0.78 (0.48–1.26) 1.16 (0.69–1.95) 1.57 (0.92–2.67)	– 1.83 (1.13–2.96)* 2.74 (1.54–4.72)*** 2.89 (1.68–5.00)***	– 1.32 (0.82–2.11) 2.06 (1.22–3.47)** 2.96 (1.71–5.11)***
Occupation						
Farmer Labour Others No-work/housewife	295 296 76 106	0.93 (0.47–1.82) 1.47 (0.74–2.91) 0.91 (0.40–2.08) –	0.50 (0.25–1.01) 1.46 (0.75–2.83) 1.76 (0.83–3.73) –	1.0152 (0.67–1.99) 1.68 (0.97–2.91) 1.37 (0.71–2.65) –	0.93 (0.50–1.70) 1.50 (0.81–2.79) 0.79 (0.38–1.66) –	0.61 (0.33–1.11) 1.16 (0.63–2.12) 1.43 (0.65–3.19) –
Caste						
ST/SC Others	538 235	– 1.41 (0.92–2.15)	– 1.07 (0.69–1.65)	– 1.23 (0.87–1.74)	– 1.22 (0.83–1.79)	– 1.67 (1.13–2.45)
Income						
< 10 K ≥ 10 K	534 239	– 0.98 (0.66–1.45)	– 0.47 (0.29–0.78)**	– 1.12 (0.79–1.56)	– 0.96 (0.67–1.39)	– 0.89 (0.62–1.28)
Wealth index						
Poorest Poorer Middle Richer Richest	154 156 154 155 154	– 0.97 (0.56–1.69) 0.93 (0.53–1.62) 0.87 (0.49–1.52) 1.12 (0.59–2.15)	– 1.18 (0.61–2.31) 0.62 (0.29–1.29) 1.128 (0.67–2.45) 0.98 (0.48–1.99)	– 0.91 (0.57–1.46) 1.12 (0.69–1.81) 1.99 (1.19–3.34)** 0.32(0.45–1.30)	– 1.03 (0.62–1.72) 0.96 (0.58–1.61) 1.20 (0.71–2.04) 1.87 (1.01–3.45)**	– 1.03 (0.62–1.69) 0.79 (0.48–1.29) 0.97 (0.58–1.61) 1.45 (0.80–2.64)

*AOR* adjusted odds ratio

***p = 0.001; **p = 0.05; *p = 1.00

Fever with chills was reported as the main symptom of malaria by the majority of the respondents. It was found that age is positively and significantly associated with the knowledge of these symptoms (AOR: 2.80, p = 0.001). Married population (AOR: 2.18, p = 0.05), better educated (AOR: 1.68, p = 0.001) and those belonging to wealthy households (AOR: 1.87, p = 0.05) were better aware of use of programme-supported preventive measures such as use of LLIN and IRS (Table 4).

Further, positive correlation was seen between levels of education and correct knowledge on malaria diagnostic tests such as RDT/blood slide examinations (AOR: 2.96, p = 0.001). The scheduled tribes and scheduled castes groups had poor knowledge of diagnostic tests for malaria (Table 4).

### Appraisal of MEDP field staff and activities

Around 87% (671) respondents were aware about the MEDP field staff working in their villages. Most of them mentioned that they could recognize the workers by name in their village. Around 89% of them replied that MEDP workers visited their houses regularly every month. Forty two percent reported that during blood testing, the workers also explained in-detail about various aspects of treatment for malaria (Table 5). Most of them (88%) mentioned about the easy understanding of the messages provided by these workers, around 98% of the respondents were satisfied with the MEDP workers and expected them to keep visiting their villages as per schedule. Most of them (87%) also expressed their willingness to extend cooperation to the workers for improvement of health communication strategies. Regarding exposure to ongoing IEC activities, 81% (546) reported to have received malaria related information during home visits by MEDP workers, 19% (125) mentioned IEC camps, and 13% (88) had witnessed both activities (Table 5).

**Table 5:** Assessment by community of Mandla towards performance of MEDP

	Frequency	Percentage
Community assessment of MEDP workers		
People know about MEDP	**671 (Out of 773)**	86.8
(Assessment made out of N = 671)		
People could recognise a village malaria worker	656	97.8
Workers visits their house regularly	597	89.0
Workers gives IEC inforation on malaria	493	73.5
During diagnosis, workers explain in details about the treatment and its importance	280	41.7
Understand the messages provided by the workers	593	88.4
Would like the worker to keep visiting them	659	98.2
People follow the learnings provided by the workers	582	86.7
People willing to help MEDP by sharing the learning to the larger groups	543	80.9
Places where malaria related information received		
House to house visits and interpersonal communication	546	81.4
Community Camp Approach	125	18.6
Both (at home and in camp)	88	13.1

Scaling technique was adopted to construct a 0–13-point score of the MEDP activities. It was observed that higher education, non-scheduled tribes, non-scheduled castes population, and those belonging to wealthy households had better knowledge and positive attitude towards MEDP activities and the association was found to be significant (Table 6). Scores were generated at 1–5-point scale for self-assessment of the respondents towards improvement in their own knowledge on malaria before and after initiation of MEDP IEC activities (Table 7). The estimated average scores revealed that after MEDP, knowledge on malaria had improved and the association was found to be significant. The self-assessed improvement was higher in cases of respondents who were aged, female, illiterate, belonged to Scheduled Tribes/Scheduled Castes, and those in lower income group households (Table 7).

**Table 6:** Knowledge and attitude about MEDP project activities (on a scale of 0–13 points)

Variables	N	Mean score (0–13 points)	ANOVA P value
Age			0.78
18–29	246	6.9 ± 3.3	
30–44	262	7.1 ± 3.2	
45 +	265	6.9 ± 3.2	
Sex			0.43
Female	256	6.9 ± 3.4	
Male	517	7.1 ± 3.2	
Marital status			0.68
Unmarried	107	7.1 ± 3.3	
Married	666	7.0 ± 3.2	
Education			0.031
Illiterate	147	6.5 ± 3.1	
Primary	188	6.7 ± 3.4	
Middle	170	7.2 ± 3.2	
High school	268	7.4 ± 3.1	
Occupation			0.02
Farmer	295	6.5 ± 3.5	
Labour	296	7.1 ± 3.1	
Others	76	7.8 ± 2.9	
No-work/housewife	106	7.5 ± 2.7	
Caste			0.16
ST/SC	538	6.9 ± 3.3	
Others	235	7.3 ± 2.9	
Income			0.000
< 10 K	534	7.33 ± 3.0	
≥ 10 K	239	6.29 ± 3.5	
Wealth index			
Poorest	154	6.4 ± 3.4	0.046
Poorer	156	7.1 ± 3.2	
Middle	154	6.8 ± 3.5	
Richer	155	7.2 ± 3.2	
Richest	154	7.5 ± 2.7

**Table 7:** Self-assessment of own knowledge about malaria (1–5-point scale) before and after initiation of MEDP IEC activities

Variables	N	Average score of knowledge (1–5 point scale)		Paired test P value
		Before MEDP	After MEDP	
Age				
18–29	246	1.6 ± 0.8	2.8 ± 1.1	0.000
30–44	262	1.5 ± 0.7	2.9 ± 1.1	0.000
45 +	265	1.5 ± 0.7	2.8 ± 1.1	0.000
Sex				
Female	256	1.4 ± 0.7	2.8 ± 1.1	0.000
Male	517	1.6 ± 0.7	2.9 ± 1.1	0.000
Marital status				
Unmarried	107	1.8 ± 0.9	3.1 ± 1.2	0.000
Married	666	1.5 ± 0.7	2.8 ± 1.1	0.000
Education				
Illiterate	147	1.2 ± 0.5	2.9 ± 1.1	0.000
Primary	188	1.4 ± 0.6	2.7 ± 1.0	0.000
Middle	170	1.4 ± 0.7	3.1 ± 1.2	0.000
High school	268	1.8 ± 0.8	2.8 ± 1.1	0.000
Occupation				
Farmer	295	1.6 ± 0.7	2.9 ± 1.1	0.000
Labour	296	1.4 ± 0.6	2.7 ± 1.0	0.000
Others	76	1.7 ± 0.8	3.1 ± 1.2	0.000
No-work/housewife	106	1.6 ± 0.8	2.8 ± 1.1	0.000
Caste				
ST/SC	538	1.5 ± 0.6	2.8 ± 1.1	0.000
Others	235	1.7 ± 0.8	2.9 ± 1.1	0.000
Income				
< 10 K	534	1.54 ± 0.7	2.9 ± 1.1	0.000
≥ 10 K	239	1.52 ± 0.8	2.8 ± 1.1	0.000
Wealth index				
Poorest	154	1.4 ± 0.6	2.7 ± 0.9	0.000
Poorer	156	1.5 ± 0.7	2.9 ± 1.0	0.000
Middle	154	1.4 ± 0.6	2.6 ± 1.1	0.000
Richer	155	1.6 ± 0.7	3.0 ± 1.2	0.000
Richest	154	1.8 ± 0.9	2.8 ± 1.1	0.000
Total	773	1.5 ± 0.7	2.8 ± 1.1	0.000

## Discussion

The primary livelihood of this low socio-economic and tribal dominated district is based on small-scale, subsistence agro-economy. The residents travel frequently and for long periods out of their home for cultivation, irrigation, construction work, fetching water, use of public toilets. Their life style exposes them to frequent mosquito bites making the population vulnerable to malaria and other vector-borne diseases. This study is similar to the one conducted in south-eastern Tanzania, where farmers spend long hours outside their residence and are exposed to mosquito bites [10]. The highly targeted IEC strategy of MEDP has been supported by existing literature, which highlights the importance of need-based health communication in improving health literacy of the community [5, 11, 12].

A clear understanding of the knowledge, attitudes and practices of a particular community is instrumental in guiding and developing Behaviour Change Communication (BCC) campaigns to achieve a stronger impact [13]. A qualitative study conducted at Gadchiroli district of Maharashtra, which is predominantly a tribal area of Maharashtra, also revealed similar misconceptions on transmission dynamics of malaria existed and act as an impediment in malaria control [8]. Another study revealed that misconceptions about causes and prevention of malaria adversely influenced the use of preventive measures against malarial infections [10]. The present study highlighted that more efforts are needed to improve the health literacy among the study population. However, it was observed that within a period of 18 months, MEDP was able to achieve significant improvement in knowledge on transmission, preventive, diagnosis and treatment related to malaria with its malaria communication campaign.

The study also revealed that programme-supported preventive measures such as IRS and LLIN were known to only 39% of the respondents. It is worth mentioning here that IRS is usually not allowed in all the rooms of the house by the community due to the fear of its ill-effects. In addition to this, most of the IRS schedule is not pre-intimated to community members, thus it does not give adequate information to prepare for household spraying programmes leading to poor acceptance. A small section of the respondents also mentioned about the misuse of LLIN. Similar observations were revealed in the Gadchiroli study [8].

The results of the study has revealed that ASHAs and ANMs are not the popular service provider for malaria diagnosis and treatment in the community, because only 18% of respondents were aware of these frontline programme driven service providers. This can be overcome by two pronged strategy: (1) Directed communication strategy to inform the community that ASHAs and ANMs are the first go-to health workers for malaria diagnosis and treatment; and (2) Subject-specific continuous training and formally adding milestone-driven service requirements as part of the job requirements of ASHAs and ANMs. The popular traditional healers could also be trained and included in the training and capacity development.

The self-assessment of the community members on the communication strategy revealed that MEDP was successful in generating significant level of awareness on malaria within 18 months of its implementation. MEDP was successful in reaching the target population, particularly in the remote areas of the district with its unique tracking, testing, treating, tracking for follow up (T4) services along with communication strategy [14]. As part of this study, we have previously documented that IEC programmes also benefited the health-workers in making them aware of the principles of surveillance, case management, and vector control [15, 16].

The study findings revealed that there was improvement in respondent’s self-reported knowledge of malaria after the introduction of MEDP’s communication strategy. Furthermore, this improvement was evident in older individuals, females, illiterates, scheduled tribes/ scheduled castes, and economically weaker population. However, the level of knowledge among them is still lower than other contemporary groups, which identifies the need for continuing focus on these vulnerable sections.

In the context of the above discussion, a prior study has revealed that there is a need for understanding the role and significance of front-line workers, qualified and unqualified practitioners, health-seeking behaviour of the community, and usage of full package of malaria commodities offered by the programme [8, 17].

## Conclusion

The present study has revealed that the communication strategy developed by MEDP in Mandla district has been useful to the local communities as they are becoming better informed on the prevention and treatment aspects of malaria. It is believed that the combination of “Pull” by the community and “Push” by the MEDP field workers has improved malaria elimination outcomes in a timely manner. A significant reduction was observed in the indigenous cases of malaria in the district and it is believed that the communication strategy has contributed significantly to the success.

## Data Availability

We have reported all the findings in this manuscript. The hardcopy data is stored at MEDP Office in Mandla, Madhya Pradesh and Indian Council of Medical Research-National Institute of Research in Tribal Health (ICMR-NIRTH), Jabalpur, Madhya Pradesh. Softcopy data is available on the project server of MEDP hosted by Microsoft Azure. If anyone wants to review or use the data, they should contact: Dr. Altaf A. Lal. Project Director – Malaria Elimination Demonstration Project, Mandla. Foundation for Disease Elimination and Control of India, Mumbai, India 482003. E mail: altaf.lal@sunpharma.com, altaf.lal@gmail.com.

## References

[1] Rajvanshi H, Bharti PK, Nisar S, Jain Y, Jayswar H, Mishra AK (2020). Study design and operational framework for a community-based Malaria Elimination Demonstration Project (MEDP) in 1233 villages of district Mandla, Madhya Pradesh. Malar J.

[2] Strategic Plan for Malaria Control in India 2012–2017. http://www.nvbdcp.gov.in/Doc/Strategic-Action-Plan-Malaria-2012-17-Co.pdf

[3] Sundararajan R, Kalkonde Y, Gokhale C, Greenough PG, Bang A (2013). Barriers to malaria control among marginalized tribal communities: a qualitative study. PLoS ONE.

[4] Saha KB, Sharma RK, Mishra R, Verma A, Tiwari B, Singh N (2015). Establishing communication mechanism for malaria prevention in Baiga tribal villages in Baiga Chak area of Dindori district, Madhya Pradesh. Indian J Med Res.

[5] Saha KB, Saha UC, Sharma R, Pandey A (2013). Reaching tribal men to improve awareness to sexual morbidities: experience from Baiga tribe of central India. Indian J Med Res.

[6] India-National Family Health Survey 1 (NFHS) 1992–93. http://rchiips.org/nfhs/india1.shtml.

[7] Jayalakshmi K (2000). Health management in the integrated tribal development agency: a case study. J Health Manage.

[8] Saha KB, Behera P, Munshi H, Tiwari BK, Singh SK, Saha UC (2019). What affects utilization of malaria control services? A qualitative approach to understanding community perception in highly malarious Gadchiroli district, Maharashtra. India J Biosoc Sci.

[9] Rajvanshi H, Saha KB, Shukla MM, Nisar S, Jayswar H, Mishra AK (2021). Assessment of ASHA for knowledge, diagnosis and treatment on malaria in Mandla district of Madhya Pradesh as part of the malaria elimination demonstration project. Malar J.

[10] Arogundade ED, Adebayo SB, Anyanti J, Nwokolo E, Ladipo O, Ankomah A (2011). Relationship between care-givers’ misconceptions and non-use of ITNs by under-five Nigerian children. Malar J.

[11] India-National Family Health Survey 2 (NFHS). https://www.dhsprogram.com/pubs/pdf/FRIND2/FRIND2.pdf.

[12] Singh N, Mishra AK, Saha KB, Bharti PK, Sisodia DS, Sonal GS (2018). Malaria control in a tribal area of central India using existing tools. Acta Trop.

[13] Mwanje LF, Comm B (2013). Knowledge, attitudes and practices on malaria prevention and control in Uganda. Trop Med Int Health.

[14] Bharti PK, Rajvanshi H, Nisar S, Jayswar H, Saha KB, Shukla MM (2020). Demonstration of indigenous malaria elimination through track-test-treat-track (T4) strategy in a Malaria Elimination Demonstration Project in Mandla, Madhya Pradesh. Malar J.

[15] Rajvanshi H, Nisar S, Bharti PK, Jayswar H, Mishra AK, Sharma RK (2021). Significance of training, monitoring and assessment of malaria workers in achieving malaria elimination goal of Malaria Elimination Demonstration Project. Malar J.

[16] Rajvanshi H, Bharti PK, Nisar S, Jayswar H, Mishra AK, Sharma RK (2021). A model for malaria elimination based on learnings from the Malaria Elimination Demonstration Project, Mandla district Madhya Pradesh. Malar J.

[17] Essé C, Utzinger J, Tschannen AB, Raso G, Pfeiffer C, Granado S (2008). Social and cultural aspects of 'malaria'and its control in central Côte d'Ivoire. Malar J.

